# Sleep Disturbances and Psychoaffective Disorders Among Nurses Working Atypical Shifts

**DOI:** 10.1192/j.eurpsy.2026.11994

**Published:** 2026-07-31

**Authors:** B. K. Jihene, B. Narjess, R. Nakhli, G. Naoures, C. Sridi, C. Farah, F. Imen, M. Maoua

**Affiliations:** 1 Clinical Psychology Unit, Occupational Medicine Department; 2 Occupational Medicine Department, Sahloul University Hospital; 3 Faculty of Medicine of Sousse, University of Sousse; 4 Laboratoire de Recherche (LR19SP03), Sousse, Tunisia

## Abstract

**Introduction:**

Sleep disorders significantly affect mental health, particularly among healthcare professionals exposed to irregular working schedules. Nurses represent a vulnerable population due to the demands of shift work.

**Objectives:**

To assess the prevalence of sleep disturbances and psychoaffective disorders among nurses working atypical schedules.

**Methods:**

A cross-sectional, descriptive-analytical study was conducted between December 2024 and January 2025 among 100 nurses at Sahloul University Hospital, Tunisia. Data collection involved a self-administered questionnaire, the Hamilton Depression Rating Scale, the Epworth Sleepiness Scale, and the Hamilton Anxiety Scale. Data were analyzed using SPSS v25, with full adherence to ethical standards.

**Results:**

A majority of participants (68%) reported sleep disturbances, and 51% perceived their work as mentally burdensome. Nearly half (47%) experienced workplace violence during their career. Most nurses (94%) reported demotivation toward their work. Depression (mild) was identified in 33% of nurses, while 35% reported mild anxiety. According to the Epworth scale, 34% showed no sleep disturbance.

**Conclusions:**

Nurses working atypical shifts experience high rates of sleep and psychoaffective disturbances, highlighting the need for preventive strategies. Interventions such as scheduled naps, individualized work schedules, and fatigue management training may promote mental health and occupational well-being.

**Disclosure of Interest:**

None Declared

